# Curation of Mental Health Recovery Narrative Collections: Systematic Review and Qualitative Synthesis

**DOI:** 10.2196/14233

**Published:** 2019-10-04

**Authors:** Rose McGranahan, Stefan Rennick-Egglestone, Amy Ramsay, Joy Llewellyn-Beardsley, Simon Bradstreet, Felicity Callard, Stefan Priebe, Mike Slade

**Affiliations:** 1 Unit for Social and Community Psychiatry Queen Mary University of London London United Kingdom; 2 School of Health Sciences Institute of Mental Health Nottingham United Kingdom; 3 Health Service and Population Research Department, Institute of Psychiatry, Psychology and Neuroscience King's College London London United Kingdom; 4 Institute of Health and Wellbeing University of Glasgow Glasgow United Kingdom; 5 Department of Psychosocial Studies, Birkbeck Institute for Social Research Birkbeck University of London London United Kingdom

**Keywords:** mental health recovery, narrative medicine, culturally appropriate technology

## Abstract

**Background:**

Mental health recovery narratives are first-person lived experience accounts of recovery from mental health problems, which refer to events or actions over a period. They are readily available either individually or in collections of recovery narratives published in books, health service booklets, or on the Web. Collections of recovery narratives have been used in a range of mental health interventions, and organizations or individuals who curate collections can therefore influence how mental health problems are seen and understood. No systematic review has been conducted of research into curatorial decision making.

**Objective:**

This study aimed to produce a conceptual framework identifying and categorizing decisions made in the curation of mental health recovery narrative collections.

**Methods:**

A conceptual framework was produced through a systematic review and qualitative evidence synthesis. Research articles were identified through searching bibliographic databases (n=13), indexes of specific journals (n=3), and gray literature repositories (n=4). Informal documents presenting knowledge about curation were identified from editorial chapters of electronically available books (n=50), public documents provided by Web-based collections (n=50), and prefaces of health service booklets identified through expert consultation (n=3). Narrative summaries of included research articles were produced. A qualitative evidence synthesis was conducted on all included documents through an inductive thematic analysis. Subgroup analyses were conducted to identify differences in curatorial concerns between Web-based and printed collections.

**Results:**

A total of 5410 documents were screened, and 23 documents were included. These comprised 1 research publication and 22 informal documents. Moreover, 9 higher level themes were identified, which considered: the intended purpose and audience of the collection; how to support safety of narrators, recipients, and third parties; the processes of collecting, selecting, organizing, and presenting recovery narratives; ethical and legal issues around collections; and the societal positioning of the collection. Web-based collections placed more emphasis on providing benefits for narrators and providing safety for recipients. Printed collections placed more emphasis on the ordering of narrative within printed material and the political context.

**Conclusions:**

Only 1 research article was identified despite extensive searches, and hence this review has revealed a lack of peer-reviewed empirical research regarding the curation of recovery narrative collections. The conceptual framework can be used as a preliminary version of reporting guidelines for use when reporting on health care interventions that make use of narrative collections. It provides a theory base to inform the development of new narrative collections for use in complex mental health interventions. Collections can serve as a mechanism for supporting collective rather than individual discourses around mental health.

## Introduction

### Background

Recovery has become a guiding ethos for mental health research, policy, and service development [1]. Recovery is defined by the individual [2] and has been described as “a way of living a satisfying, hopeful and contributing life whether or not the limitations of illness continue” [3]. A focus on recovery extends the traditional clinical priority of symptom amelioration to a more holistic perspective on mental health [4,5]. The development of an associated recovery movement has centered on the experience of the individual [6] and emphasizes the importance of including knowledge from experts by experience in understanding mental health problems [7]. This orientation places an increased emphasis on first-person knowledge and encourages provision of care to be tailored to the individual [8].

An emphasis on mental health recovery can lead to an increased use of recovery narratives. For the purposes of this paper, a recovery narrative is defined as a first-person lived experience account of recovery from mental health problems, which refers to events or actions over a period [9] and which can be given live or in a recorded form [10]. Live recovery narratives are shared in the context of an in-person or Web-based relationship and involve some form of mutual exchange, whereas recorded recovery narratives are presented in an invariant form, frequently as text, audio, or video but occasionally in formats such as visual artworks [11]. Access to both categories of narrative is increasing [10], and a recent systematic review has identified both helpful and harmful impacts that they can have on recipients [10].

Recovery narratives are regularly used as a resource in health care practice [12]. At the level of public health, recovery narratives have been used as an effective resource in antistigma campaigns [13,14], where they can act as a form of social contact between people with experience of mental health problems and others [15]. Written recovery narratives are a useful resource in psychotherapy sessions [16,17], and a US national survey has shown that the sharing of live recovery narratives is a key feature of the work of peer specialists [18]. The sharing of live recovery narratives is also a feature of recovery education approaches such as recovery colleges [19], where they might be developed through *Telling My Story* courses, which can provide benefits to both the narrators and recipients [20]. More broadly, the relevance of narratives to mental health is well established; there is a consensus that their creation and consumption can be helpful both to the individual sharing their story and to the intended recipients [21]. Indeed, traditional talking therapies have been likened to a process of joint narrative creation [22], and case histories have long been used to educate health care professionals [23].

When recovery narratives are presented in a recorded form, they can be grouped into collections, and the emergence of a wide range of publicly available collections of recovery narratives is a notable phenomenon of at least the last 20 years. Early examples of collections were published in books, often grouping together narratives sharing a common diagnosis or symptomatology [24,25], sometimes explicitly motivated by intentions such as presenting a positive outlook for people experiencing mental health problems or for carers [25]. Collections of written recovery narratives have also been presented in health service booklets, which are intended to make real stories of recovery available to other service users [26], and in regular series of personal accounts in academic journals such as *Psychosis* [27] and *Psychiatric Services* [28]. Collections of recorded recovery narratives are also widely available on the Web, sometimes presented in bespoke websites, where they might be explicitly motivated by their value as a stigma reduction tool [29] or as a reference resource for people experiencing mental health problems [30]. Other forms of Web-based collection include series of video or audio blogs hosted by charities [31,32], providing a moderated route for people to share their recovery experiences.

Creating and disseminating collections of recovery narratives require individual or collective effort in addition to the effort of producing the individual narratives included in a collection. Examples might include locating potential contributors or selecting and organizing submissions. In this paper, the work done to enable a collection is referred to as *curation*, and the people who do it are referred to as *curators*. Our usage of this term draws on existing usage within the discipline of museum studies, where the work of curators has been studied and taught for several centuries [33] and is understood as both a purposeful and political act, with curators often engaging with artifacts or collections that are sensitive and challenging [34]. The study and teaching of curation focus on reflective practice as a mechanism for understanding how to negotiate sensitivities and to bring meaning to the artifacts being curated [35]. Recently, digital curation has adopted as a term to cover the long-term management of digital data [36] and has also emerged as a research topic in its own right [37].

There are a range of specific sensitivities around recovery narratives, and we might expect the work of curating recovery narrative collections to be sufficiently different from that of curating a museum exhibit that it is worthy of study. Recovery narratives can contain sensitive and personal information such as experiences of distress and criticisms of interactions with health services. They can identify the narrator as well as third parties, which is important given the ongoing existence of stigma in relation to mental illness [38]. Similar to other forms of health material, engaging with the content presented in a recovery narrative may cause emotional distress on the part of recipients [39] and have adverse effects on people accessing the narratives [40], particularly given the known risks around receiving Web-based material and self-harm [41]. Curators shape and influence the material that is presented and, hence, what is available for usage by others, and this is particularly relevant given the contested nature of recovery as a concept [42] and the status of recovery narratives as tools of resistance, opposition, collective action, and social change [43].

In building collections, curators are likely to have developed specific knowledge and practices to address these issues, and given an increasing use of recovery narratives in health care practice, developing a systematic understanding of decisions made by curators might provide benefits to their future health services usage. It might also provide a greater understanding of the characteristics of recovery narrative collections used in health service practice, such as biases in the composition of these collections affecting the types of narrative included.

### Aims of the Study

No previous systematic review on the curation of mental health recovery narratives has been conducted. The aim of this study was to produce a conceptual framework identifying and categorizing decisions made in the curation of mental health recovery narrative collections.

## Methods

### Design

A systematic review was conducted to identify and synthesize documentary information on curatorial decision making for collections of mental health recovery narratives. The systematic review followed guidance provided in the PReferred Reporing Items for Systematic reviews and Meta-Analyses (PRISMA) statement [44]. A protocol for the search and synthesis process was published in advance through PROSPERO [45]. Pilot searches of bibliographic databases identified minimal peer-reviewed empirical research evidence on this topic and hinted at a gap in research knowledge, and hence, the review was designed to enable the creation of a preliminary conceptual framework to inform the design of future research work. To produce an informative framework, searches were designed to systematically locate all available peer-reviewed research articles and to locate a selection of nonresearch documents produced by curators, providing direct evidence on curatorial decision making.

A qualitative evidence synthesis was conducted of all sources while retaining a clear audit trail of concepts derived from included peer-reviewed research articles. Documents were included in the synthesis if (1) they related to a collection containing at least three recovery narratives about mental health (any diagnosis or mix of diagnoses, excluding solely substance abuse), (2) the document contained researcher- or curator-derived information about decision making around the curation of the collection, (3) the document was published in English, (4) the publication date was before July 31, 2018, and (5) the collection was publicly available (on the Web or in print).

### Data Sources

#### Publications in Bibliographic Databases

Research on recovery narratives is interdisciplinary [12], and hence, a broad range of bibliographic databases were searched to identify peer-reviewed publications. The selection of these databases was informed by database selections in 2 parallel systematic reviews on the characteristics [12] and impact of recovery narratives, augmented by scoping searches and expert consultation. A total of 13 bibliographic databases were searched from inception to July 31, 2018: Applied and Complimentary Medicine Database, Applied Social Science Index and Abstracts, Cochrane Library, CINAHL, EMBASE, JSTOR, MEDLINE, PsycARTICLES, PsycINFO, Scopus, Social Science Research Network, Web of Science, and ACM digital library. The search was designed as shown in Textbox 1 and was specialized to each of the relevant databases as needed. Scoping searches were used to select a range of synonyms for use within each clause.

**Bibliographic database search.**

(Curat* OR Manag* OR creat* OR oversee* OR assembl* OR collect* OR present*)

AND

(psych* health OR psych* illness OR psych* problem* OR psych* disorder OR mental distress OR emotional distress OR recover* OR trauma OR Mental* OR psych* OR mad OR madness OR emotional distress OR trauma)

AND

(narrative* OR stories OR account* OR experience* OR tale* OR lived experience OR personal experience OR testimon*)

AND

(repositor* OR collection* OR compendi* OR antholog* OR forum OR blog OR vlog)

Searches were conducted by RM and AR, and the resulting papers were collated by RM, who removed duplicates. RM and AR screened the titles and abstracts of the remaining papers according to the inclusion criteria to identify those that were potentially eligible. RM and AR independently rated 1 in 5 of the other’s screening for consistency, achieving complete concordance. The full-text versions of the remaining papers were screened for eligibility by RM. AR independently rated 1 in 5 for consistency, achieving complete concordance.

#### Specific Journals

Overall, 3 journals (*Schizophrenia Bulletin, Psychosis,* and *Psychiatric Services*) were identified as regular publishers of recovery narratives. Journal indexes were hand searched from inception for peer-reviewed publications, and journal websites were hand searched for nonresearch documents.

#### Gray Literature Databases

Variants of the search terms outlined above were used to search the gray literature for peer-reviewed publications and nonresearch documents using dissertation database searches, Google Scholar, BASE, and OpenGrey.

#### Forward and Backward Citation Tracking

Forward citation tracking of included peer-reviewed publications was conducted using Google Scholar. The reference lists of included peer-reviewed publications were hand searched.

#### Digitally Accessible Books

Scoping searches had shown that printed books presenting collections of recovery narratives sometimes began with an editorial chapter providing information about how the book had been curated. A sample of books was identified using Google Books, a large Web-based repository of digitized texts estimated to index more than 30 million books [46]. Google Books offers a relatively restricted searching interface. Guided by scoping searches, the synonymous search term “mental health recovery stories” was used to search Google Books. The 50 books that came up in the first 5 search pages of search results were accessed and hand searched for the presence of an editorial chapter. If present, this chapter was treated as a candidate documentary source and was assessed against the inclusion criteria.

#### Web-Based Collections

Web-based collections identified using the search term “mental health recovery stories” in the Google search engine were hand searched for nonresearch documents. Collections identified in the first 5 search pages were hand searched.

#### Health Service Booklets

Scoping searches demonstrated that health service booklets can contain editorial sections presenting information on curation, but they also identified that these booklets are frequently not available on the Web or in publication databases. Health service experts (n=7) were consulted for recommendations of specific health service booklets that included information about the process of curating the booklets. Editorial sections of recommendations were treated as a candidate documentary source and were assessed against the inclusion criteria.

### Quality Assessment

A quality assessment of all included peer-reviewed qualitative research publications was conducted using the Critical Appraisal Skills Programme (CASP) qualitative checklist [47], using an established scoring system and thresholds for high-, moderate-, and low-rated quality (high: 9-10, moderate: 7.5-8.5, and low: 0-7) [48]. The rated quality did not determine inclusion. No quantitative research publications were included.

### Data Extraction and Synthesis

Short narrative summaries were produced of included qualitative research publications and are included in the Results section. These summarize the methods, rated quality, and curatorial issues identified in the publication.

Owing to a lack of previous frameworks on the curation of mental health recovery narratives, a qualitative synthesis [49] of all included documents was conducted using inductive thematic analysis. In stage 1, text present in 1 research article [50] and 2 contrasting documents relating to Web-based collections [51,52] was analyzed by RM and SRE to identify preliminary curatorial themes that are presented in Multimedia Appendix 1. In stage 2, a thematic analysis of all the included documents was conducted. The included documents and preliminary themes were transferred into NVivo version 11 (QSR International) for data handling and analysis by RM and SRE. The relevant material in documents was coded, and initial themes were extended and restructured into hierarchies through constant comparison [53]. In stage 3, this framework was refined into a conceptual framework by a broader analysis team that included experts in recovery research, digital curation, and health sociology and an experienced curator of a recovery narrative collection. The names of themes included in the framework were refined, and some included subthemes mere merged. Contributions from text fragments coded in the included research publications were tracked so that the contribution to the conceptual framework of research publication could be highlighted in the Results section.

Once the final conceptual framework had been established, planned subgroup analyses were conducted on (1) documents relating to Web-based collections and (2) documents relating to printed collections. These were used to compare and contrast the relative strengths of conceptual framework themes in these 2 subgroups.

## Results

### Flow Diagram

**Figure 1:**
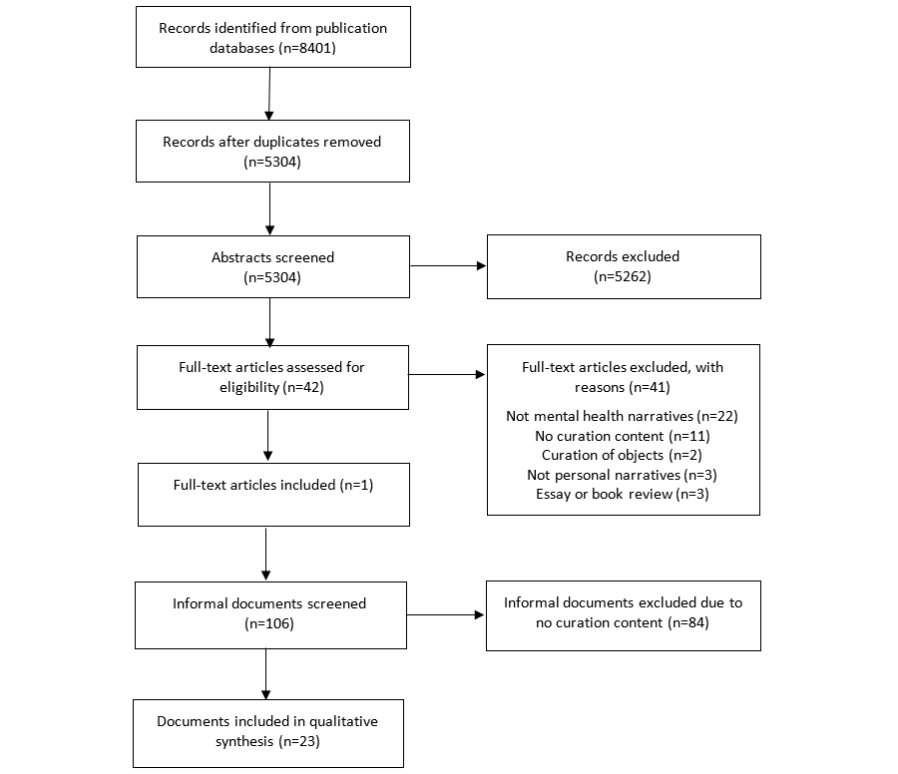
Preferred Reporting Items for Systematic Reviews and Meta-Analyses (PRISMA) flow diagram for documents included in qualitative synthesis.

The PRISMA flow diagram for the systematic review is shown in Figure 1.

### Summary of Included Documents

The 23 documents included in the qualitative evidence synthesis are summarized in Table 1. Each has been assigned unique identifiers (UIDs). These UIDs are listed in Table 1, and are referred to in the samples of coded text presented in Multimedia Appendices 1 and 2. Table 1 also includes references to all included documents, to enable replication, and to provide researchers with a corpus of publicly available documents with insights into curation.

One peer-reviewed article was included [50]. The other 22 documents comprised documents providing descriptions of how curation had been structured for specific Web-based collections (n=4), Web-based documents written to provide guidance for narrators wishing to submit material to specific collections (n=11), editorial book chapters (n=4), and forewords to health service booklets of mental health recovery narratives (n=3). For 2 of the Web-based collections, hand searching of websites identified 2 includable documents each, and hence, both were included in the synthesis. None of the included booklets were indexed in publication databases or search engines.

**Table 1:** Summary of documents subject to inductive thematic analysis.

UID^a^	Reference	Categorization	Collection(s) referred to	Country of collection	Narrative format
1	Crossley and Crossley [50]	Journal article	Two books: The Plea for the Silent [54] and Speaking our Minds [55]	England	Text
2	Health Talk [51]	Description of curation	HealthTalk: Web-based collection of health narratives	England	Audio, text, and video
3	Bradstreet [56]	Description of curation	Multiple collections of mental health recovery narratives curated by the Scottish Recovery Network	Scotland	Audio, text, and video
4	Write to Recovery [57]	Description of curation	The “Write to Recovery” Web-based recovery narrative collection	Scotland	Text
5	Time to Change [52]	Guidelines for narrators: Web-based collection	Time to Change collection of mental health blogs	England	Text
6	Mind, the Mental Health Charity [58]	Guidelines for narrators: Web-based collection	Mind collection of blogs and vlogs	England	Text and video
7	NAMI California [59]	Guidelines for narrators: Web-based collection	National Alliance on Mental Illness collection of “Share your story” blogs	United States	Text
8	NAMI - You are Not Alone [60]	Guidelines for narrators: Web-based collection	National Alliance on Mental Illness collection of “Share your story” blogs	United States	Text
9	Empower Idaho [61]	Guidelines for narrators: Web-based collection	Empower Idaho collection of recovery stories	United States	Video
10	Crowe [62]	Description of curation	Australian Government National Mental Health Commission collection of “Personal stories”	Australia	Text
11	Resources to Recover [63]	Guidelines for narrators: Web-based collection	Resources to Recovery collection of “Stories of hope and recovery”	United States	Text
12	Boll [64]	Guidelines for narrators: Web-based collection	Resources to Recovery collection of “Stories of hope and recovery”	United States	Text
13	Substance Abuse and Mental Health Services Administration [65]	Guidelines for narrators: Web-based collection	Various collections curated by the Substance Abuse and Mental Health Services Association	United States	Text
14	Mental Health Stories [66]	Guidelines for narrators: Web-based collection	Mental Health Stories collection of recovery stories	England	Text
15	LeCroy and Holschuh [67]	Editorial chapter of a book	Book with title “First-person accounts of mental illness and recovery”	United States	Text
16	Gilbert [68]	Editorial chapter of a book	Book with title “Beating Depression: Inspirational Stories of Hope and Recovery”	England	Text
17	Basset and Stickley [69]	Editorial chapter	Book with title “Voices of Experience: Narratives of Mental Health Survivors”	England	Text
18	Gray [70]	Editorial chapter	Book with title “The Madness of Our Lives: Experiences of Mental Breakdown and Recovery “	United States	Text
19	International Mental Health Collaborating Network [71]	Foreword to booklet	Booklet with title “Recovery Stories: Cornish Journeys of Hope”	England	Text
20	CMHT Institute of Mental Health [72]	Foreword to booklet	Booklet with title “Journey to Recovery”	Singapore	Text
21	South London and Maudsley NHS Foundation Trust [26]	Foreword to booklet	Booklet with title “Moving Forward: Stories of Recovery”	England	Text
22	Oxford University Press: Schizophrenia Bulletin [73]	Guidelines for narrators: academic journal	Schizophrenia Bulletin: Collection of “first person accounts”	United States	Text
23	Psychiatry Online [74]	Guidelines for narrators: academic journal	Psychiatric Services: Collection of “personal accounts”	United States	Text

^a^UID: unique identifier.

### Narrative Summaries of Findings in Included Peer-Reviewed Articles

The single included peer-reviewed paper [50] presented a comparative analysis of 2 books presenting collections of narrative identifiable as recovery narratives within the definition adopted for this review. This paper was rated to be of moderate quality using CASP.

The 2 books considered in this paper were *The Plea for the Silent* [54] and *Speaking our Minds* [55], published in 1957 and 1996, respectively. Through this analysis, key curatorial considerations were identified as (1) intended societal influence of the collection, (2) approach to narrator safety, and (3) approach to establishing authenticity of included narratives. The paper explains that *The Plea for the Silent* was published in a society with high levels of legal and social discrimination against people with experience of mental health problems, with an explicit purpose of enacting change but also a need to protect contributors. To support influence on society, curators chose to justify the authenticity of narratives with reference to formal health records and the perceived societal status of the narrators (eg, by stating that narrators were civil servants or teachers). They chose to anonymize the narrators to protect them from stigma or legal difficulties. *Speaking our Minds* was published into a society with higher levels of activism around the rights of people experiencing mental health problems. The included narrators were all activists. The curators chose to name narrators so as not to deny them a personal voice and to justify authenticity in relation to activism activities.

### Conceptual Framework on the Curation of Mental Health Recovery Narratives

The conceptual framework derived through inductive thematic analysis is presented in Table 2. It identifies 9 higher level curatorial issues present in included documents, each of which is accompanied by subthemes identifying more specific curatorial issues. Each subtheme is illustrated with short textual descriptions of specific choices adopted by curators. Choices were included if they were identified in 1 or more source documents. Choices identified through the included peer-reviewed article [50] are highlighted in italics, and all other choices have been identified from informal documents. For the latter, illustrative text coded against that choice is provided in Multimedia Appendix 2.

In some cases, the range of choices structured within a theme highlights contentious issues where a curator has to pick from a range of competing possibilities. One example is anonymization as a route to narrator safety, where 3 alternatives are present (anonymize narrators to protect identity, clearly identify narrators to give them a voice, and provide guidance on choices around revealing narrator identity).

### Strength of Theme Analysis: Curation in Web-Based and Printed Collections

The conceptual framework integrates curatorial issues and choices across Web-based and printed collections, where printed collections were composed of published books and health service booklets. Subgroup analyses were used to identify issues or choices which were more relevant to either (1) Web-based or (2) printed collections.

Providing benefits for narrators as well as recipients was more of a concern for Web-based collections. This was because of the open-ended and interactive nature of Web-based collections relative to published books, meaning that they often encouraged recipients to submit their own stories and, hence, become narrators themselves. Audience interaction was also more relevant to Web-based collections.

Societal positioning as a superordinate category was commonly found in printed books where it appeared in explicit editorial reflections on the context in which the book was published, provided to offer explanation and justification for the stance taken in curating the material presented in the book.

A theme that emerged as important across all collections was safety of the narrator, recipient, and third parties mentioned in narratives. Recipient safety was discussed more frequently in relation to Web-based collections, presumably because of the instantly accessible nature of Web-based material, making it more likely to be accessed by people experiencing severe distress.

Online calls for submission were much more common for Web-based collections and typically led to direct submission of narratives rather than the construction of narratives through interviewing. The process of selecting narratives was not discussed in any depth in any included documents.

How to edit material emerged as an important issue for curators of all collections (eg, it was discussed in many documents). It was split into editing to support narrator and third-party safety and editing for clarity of presentation. No mention was made of the legal implications of editing and, hence, becoming potentially responsible for content.

Ordering of narratives was more relevant to printed material, presumably because of its inherently linear presentation. Allowing a diversity of formats was more relevant to collections presented on the Web, reflecting the greater freedom of online presentation.

**Table 2:** Conceptual framework of curatorial issues and choices.

Curatorial issues and specific choices^a^			UID^b^
**Purpose**			
	**Narrator benefits**		
		To support narrators’ recovery	13
		To empower narrators	13,17
	**Recipient benefits**		
		To help recipients understand mental health problems	15
		To help recipients talk about mental health problems	15
		To help recipients understand when to seek help	12
	**Societal influence**		
		To reduce stigma about mental health	12
		*To provide access to unheard voices*	1
**Audience**			
	**Identification**		
		Target people with an interest in mental health	20
	**Interaction**		
		Allow commenting on narratives	6
**Safety**			
	**Narrator safety**		
		*Anonymize narrators to protect identity*	1
		*Clearly identify narrators to given them a voice*	1
		Provide guidance on choices around revealing narrator identity	8,3
		Develop a supportive relationship with a narrator	2
		Provide guidance on the emotional impact of creating narratives	13
		Provide guidance on how sharing might impact relationships	3
		Signpost narrators to resources that can help if distressed	8
		Continue to support a narrator after a narrative is public	3
	**Recipient safety**		
		Provide guidance to narrators on how to create narratives that exclude features known to trigger harmful behaviors	6
		Moderate comments in narratives shared on the Web	6
	**Third-party safety**		
		Provide guidance on protection of others identifiable in narratives	3
**Collection of narratives**			
	**Recruiting narrators**		
		Targeted requests (through health services, support groups, targeted advertising)	2,22
		Online calls for submission (on organizational websites)	7,8
	**Creation of narratives**		
		Interviews with narrators	2
		Direct submission by narrators	7,8,12
**Selection of narratives**			
	**Narrative selection**		
		Review submitted material	8
	**Narrative diversity**		
		Seek a diverse range of narratives	3
**Editing of narratives**			
	**Editing for clarity**		
		Shorten, enhance flow, and remove repetition	18
	**Editing for safety**		
		Destroy identifying information	21
**Presentation of narratives**			
	**Ordering**		
		Order narratives by clinical diagnosis	3
		Order narratives to highlight mutual connections	18
	**Format**		
		Allow a diversity of formats	3
		Present narratives that conform to a specific format	11
	**Authenticity**		
		*Established through references to formal health records*	1
		*Established through reference to societal status of narrator*	1
		*Established through reference to narrator activism*	1
**Ethics and legality**			
	**Consent**		
		Establish clear consent for use (written or verbal)	3
	**Ownership**		
		Establish through formal written agreements	7
	**Societal positioning**		
		Position relative to public policy	17
		Position relative to clinical language	3

^a^Italicized text indicates a choice identified from a peer-reviewed article.

^b^UID: unique identifier.

## Discussion

### Principal Findings

This review revealed a lack of empirical research into the curation of collections of mental health recovery narratives, with only 1 peer-reviewed paper located from the extensive database search. This is a significant result, given the ongoing public health usage of such collections (eg, in antistigma campaigns) and the influence that curators will have on the content and presentation of collections and, hence, potentially, on how mental health issues are perceived by recipients of narratives presented in collections.

The review demonstrates that documentary information about the curation of mental health recovery narrative collections does exist, mostly presented alongside the collections themselves, with relevant information sometimes distributed across multiple documents. This observation enables further work drawing on such documents as an evidence source, for example, to provide further insights into considerations unique to a specific type of collection such as a health service leaflet.

A conceptual framework of 9 major curatorial issues was identified from publicly available documents: purpose, audience, safety, collection of narratives, selection of narratives, editing of narratives, presentation of narratives, ethics and legality, and societal positioning. This provides an evidence-based foundation for future research to establish good practice guidelines for the curation of collections as they increase in number and reach. It could serve as an interim guide to issues that curators of new or existing collections should consider when deciding how to structure their work. It could be used as a preliminary version of reporting guidelines for health care interventions that make use of narrative collections.

Curation in museums studies has been introduced as both a *purposeful* and a *political* act [34], and this was reflected in specific knowledge about the curation of mental health recovery narratives developed through this review. Some collections had clearly been created for a specific purpose. Identified purposes were recognizably specific to mental health, focusing on either supporting or enhancing the mental health of individuals (narrators and recipients) or creating a healthier society. Political issues considered by curators included the relationship of the collection to public policy positions at the time of curation and clinical language as a contentious issue, especially in relation to its use to present and order narratives.

The review highlighted safety (of narrators, recipients, and third parties) as an important curatorial issue. It revealed a lack of consensus around anonymization as a route to safety, which was reflected in a tension between an approach of obscuring identity and hence protecting a narrator or third party from damaging responses such as stigma and empowering narrators by allowing them to be identifiable and hence giving them a recognizable voice. A middle way, of supporting choice by providing narrators with guidance on how to make choices about their identity, was present. A lack of consensus around issues of safety may indicate that there is no *best* curatorial approach to this issue. Rather, curators may benefit from awareness of a range of strategies to select from.

The processes of selection and presentation of narratives were identified as places in which the emergent properties of groups of narratives were actively considered by curators. In some cases, curators of collections were explicitly interested in assembling a diverse set of experiences, while still respecting the individual rights of contributors [75]. This suggests the value of using collections of narratives, rather than individual narratives, in health care practice.

### Limitations

The review only included publicly available documents. Their accuracy in reflecting decisions made around curation cannot be verified, and they may not provide complete information about all curatorial decisions made as curators may not discuss some decisions publicly. In-depth interviews with curators of collections might augment the conceptual framework developed for this review.

Although the breadth of database searches means that all available research evidence has been included, for feasibility, the review placed limitations on the number of search engine pages used to identify informal documents. Therefore, the presented framework does not draw on all available nonresearch documents. The conceptual framework might be extended by a review that considered evidence presented in all printed works or in all Web-based collections. Such a review may need to consider thousands of collections.

### Comparison With Previous Work

If some curators are explicitly interested in the use of collections of recovery narratives to present a more holistic view of mental health problems, then the work of a curator might be seen as intersecting with the emerging discipline of Mad Studies [76], which has an interest in how to general collective discourses about experiences of mental ill health and its relationship to unhealthy aspects of society is. Curating collections might be seen as loosely analogous to the Mad Studies concept of “centralizing of experiential knowledge,” described by Sweeney [77]. Curators of mental health recovery narratives might then be seen as activists, and this view is certainly present in museums studies where curatorship has been positioned as a form of social practice [78] and where approaches to the curation of culturally sensitive material, such as indigenous remains [79], homophobia [80], or damaging working conditions in sweatshops [81], have been selected to draw attention to societal problems. Contributing a narrative to a collection may also be seen as a form of activism, and digital research into affinity spaces highlights the social capital that an individual may develop through contributing a narrative [82]. The latter provides further insights into the dangers of anonymizing narrators (in that enforced anonymization precludes the use of a narrative by its narrator to develop social capital).

The safety of narrators, recipients, and third parties emerged as an important topic for curators. As such, choices identified in the review might be seen as part of a wider body of research around the safe usage of online health material, which might in turn inform curatorial practices. A realist review [40] has documented the exposure to contradictory or misleading health material as a route to harm, and this might be seen as a rationale for curatorial work to establish the authenticity of contributors A literature review has documented mechanisms by which online health material can trigger self-harm or suicide, and how to handle this kind of material might be thought of as a key consideration in recipient safety [41]. Our review has located choices around audience participation as a part of the curatorial process and the use of moderation of potential comments by curators as a possible tactic. The use of moderation has been considered in a previous review about online peer support, where the majority of interventions that were reviewed included some form of moderation by health care professionals, researchers, or service users [83]. In some cases, interaction without moderation can have harmful consequences for both people who are sharing and those who are receiving the material [84]. However, mental health issues such as stigma are regularly discussed in nonmoderated Web-based interfaces such as Twitter [85].

From an ethical perspective, recovery narrative collections have the potential to both benefit and harm others, so the biomedical ethical principles of beneficence and nonmaleficence are relevant [86]. Future research to investigate the types and mechanisms of impact is needed, and evaluations of the use of recovery narratives should specifically consider potential harms [87].

### Conclusions

This review has presented a conceptual framework identifying issues that curators of mental health recovery narrative collections attend to, drawing on available research publications and other public documents. This framework might be used to inform good practice guidelines for narrative curation and as a preliminary version of reporting guidelines for use when reporting on health care interventions that make use of narrative collections. Our study has highlighted the role of curators in shaping the material that they present and, hence, in shaping an understanding of mental health issues in recipients. Further work might extend this conceptual framework through interviews with curators so as to access details about decision making that are not available in public documents. It might also examine the impact of curatorial decisions on recipients of narrative collections.

## Appendix

Multimedia Appendix 1 Curatorial issues emerging from initial joint analysis of a sub-sample of 3 (13%) of 23 included documents.

Multimedia Appendix 2 Samples of text coded against items in the conceptual framework in included informal documents.

## Abbreviations

CASP: Critical Appraisal Skills Programme
NIHR: National Institute for Health Research
PRISMA: PReferred Reporing Items for Systematic reviews and Meta-Analyses
UID: unique identifier
