# Supplementary material for: Curation of Mental Health Recovery Narrative Collections: Systematic Review and Qualitative Synthesis
Source: JMIR Ment Health. 2019 Oct 4;6(10):e14233. doi: 10.2196/14233 (PMC6915799; doi:10.2196/14233)
Supplement: Multimedia Appendix 1 [file mental_v6i10e14233_app1.pdf]

Multimedia Appendix 1. Curatorial issues emerging from initial joint analysis of a sub-sample of 3 (13%) of 23 included documents

**Curatorial theme**	**Sample text coded in the source document (ID of source document in brackets)**
1. Preparation	Before the researchers start interviewing, they read published books and papers on the subject to help identify issues for the interviews and to find out what sorts of people we need to include in the study. Their reading includes up-to-date reviews of clinical evidence on the particular topic as well as social science studies, and will include recommendations from the advisory panel. (2)
2. Purpose	Personal blogs, vlogs and stories can be a powerful way to change attitudes. By sharing your story, you can spread knowledge and perspective about mental illness that could change the way people think about it. (5)
3. Interview process	The interviews take place throughout the UK, often in peoples' homes but people can be interviewed elsewhere if they prefer. We usually interview people on their own, but sometimes a partner or friend may be present during the interview. All the interviews are audio tape recorded for analysis. If the respondent is happy to consent, interviews are also filmed. (2)
4. Legal, ethical, and copyright issues	After reviewing the transcript (if they wish to) the interviewee is asked to sign a copyright form (to the University of Oxford) which allows the interview to be used for research, teaching, broadcasting and on the website. (2)
5. Use of repository data	We aim to publish one or two papers in peer reviewed journals from each of the studies. These are sent to social science and specialist and general medical journals. Over 60 articles have been published since 2001. We regard this as an important way of disseminating our results widely to all types of professional readers as well as ensuring that the quality and academic credibility of the studies is maintained. (2)
6. People	People involved in repository curation
6.1 Interviewers, researchers	Each researcher is fully trained in the research process, and is given a detailed handbook to refer to. The research director has overall responsibility for ensuring the research process is adhered to and approving any changes to it, and conducts annual appraisals of each researcher. The full research team meets together every 4-6 weeks to monitor progress with the individual studies, provide mutual help and advice, and deal with any questions or problems. A particular strength of the process is that each researcher is assigned another researcher who acts as ‘buddy’. The buddy supports and advises their colleague at all stages. (2)
6.2 Participants, narrators	To make sure that a wide range of experiences and views are included we use a method called purposive (or maximum variation) sampling (Coyne, 1997). We carry on collecting interviews until we are convinced that we have represented the main experiences and views of people within the UK. Often this requires between 40 and 50 interviews. (2)
7. Style of content	Finally, the summaries will be given a final edit for ‘plain English’. The research team are given regular refresher training in writing for a lay audience. (2)
8. Topic Matter, Content	The researcher identifies video, audio and written excerpts from the interviews to illustrate the range of views and experiences in the interviews. Only a few clips from each interview appear on the site, however the full interviews are used for the analysis and as the basis for papers for conference presentations, peer reviewed journals and chapters. (2)
9. Context	It might be argued that these initial differences between the two texts can be understood in terms of context. The contributors to the 1957 text simply could not speak as the contributors to the 1996 text do because they would not have been listened to or taken seriously. (1)
