# Supplementary material for: Curation of Mental Health Recovery Narrative Collections: Systematic Review and Qualitative Synthesis
Source: JMIR Ment Health. 2019 Oct 4;6(10):e14233. doi: 10.2196/14233 (PMC6915799; doi:10.2196/14233)
Supplement: Multimedia Appendix 2 [file mental_v6i10e14233_app2.pdf]

Multimedia Appendix 2: Samples of text coded against items in the conceptual framework in included informal documents.

1. Purpose		
1.1 Narrator benefits	To support narrators' recovery	The process of sharing one's recovery experience can be a healing and empowering exercise for storytellers, as well. (13)<br>Why should you share your story? Because: … It can be a healing and empowering experience for you, too. (12)
	To empower narrators	It has been our intention to create a platform for people's voices to be heard. When people have a voice, they have power. (17)
1.2 Recipient benefits	To help recipients understand mental health problems	Our hope is that this book will inform readers about mental illness/mental disorder… (15)
	To help recipients talk about mental health problems	Our hope is that this book will … help instructors, students, and the general public talk about their experiences with it whether a client's, their own, a family member's, or a close friend's. (15)
	To help recipients understand when to seek for help	Why should you share your story? …<br>It may encourage others to seek help … (12)
1.3 Societal influence	To reduce stigma about mental health	Why should you share your story? Because:<br>It helps to reduce negative attitudes and stereotypes (12)
2 Audience		
2.1 Identification	Target people with an interest in mental health	This book should be read by all with an interest in mental health. (20)
2.2 Interaction	Allow commenting on narratives	A blog post starts a conversation and invites others to comment and share their own experiences. Please remember to be courteous to your commenters, and to assume that their questions are asked in good faith. All comments are moderated, but if you have any concerns about them, don't hesitate to contact us. (6)
3 Safety		

3.1 Narrator safety	Develop a supportive relationship with a narrator	The discussions prior to story sharing provide the foundations for a supportive relationship. The process of investing time to create a document, or spoken agreement, gives value to the experience for all involved. (2)
	Provide guidance on choices around revealing narrator identity	Please note: Submissions will include your name as entered. Please DO NOT use your last name if you do not want your full name to be searchable. If you would like for your post to be anonymous, do not include your name in the body of the submission. (8)<br><br>You can use an alias or pseudonym and perhaps refrain from giving identifiable information in order to protect your identity … (3)
	Provide guidance on the emotional impact of creating narratives	Maintaining wellbeing: Planning, including exploring triggers and coping strategies, can assist you in maintaining your wellbeing throughout the narrative sharing process. (13)
	Provide guidance on how sharing might impact relationships	Cultural issues: An awareness of what mental health and wellbeing means within an author's community is important. For example, in some BME communities a person's identity may be closely linked with their place in their family and community. This may affect the way in which you support their story sharing. (3)
	Signpost narrators to resources that can help if distressed	While we have created [repository] to be an open, safe place to express yourself, if you or someone you know needs immediate help, please contact [hotline telephone number]. (8)
	Continue to support a narrator after a narrative is public	Support is still important after publication or sharing especially if the narrative draws further media attention. Ensure the author has your current contact details and arrange a follow up meeting or telephone appointment for review and support purposes. (3)
3.2 Recipient safety	Provide guidance to narrators on how to create narratives that exclude features known to trigger harmful behaviours	We also avoid publishing triggering descriptions of suicide or self-harm. Please read our guidance for blogging about self-harm, suicide, and eating disorders so that you can share the story of your experience with these topics in a way that's safe for readers. (5)
	Moderate comments in online narratives	A blog post starts a conversation and invites others to comment and share their own experiences. Please remember to be courteous to your commenters, and to assume that their questions are asked in good faith. All comments are moderated, but if you have any concerns about them, don't hesitate to contact us. (6)
3.3. Third party safety	Provide guidance on protection of others identifiable in narratives	You can use an alias or pseudonym and perhaps refrain from giving identifiable information in order to protect your identity … and the identity of others featured in your stories. (3)
4 Collection of narratives		

4.1 Recruiting narrators	Targeted requests[through health services, support groups, targeted advertising]	We recruit people to take part in interviews through a number of different routes including GPs and hospital consultants, support groups and newsletters, advertising in the press, on websites, in social media and by word of mouth. (2)<br><br>We welcome other contributions from patients, ex-patients, or family members… Clinicians who see articulate patients, with experiences they believe should be shared, might encourage these patients to submit their articles to *[name of journal section]*, (22)
	Online calls for submission [on organizational websites]	By submitting a post for publication on [charity]'s blog [7]<br><br>Every story submitted is reviewed by NAMI and all stories may not be posted on the blog. (8)
4.2 Creation of narratives	Interviews with narrators	We recruit people to take part in interviews … (2)
	Direct submission by narrators	By submitting a post for publication on [charity]'s blog [7]<br><br>Every story submitted is reviewed by NAMI and all stories may not be posted on the blog. (8)<br><br>Why should you share your story? Because:<br>It helps to reduce negative attitudes and stereotypes,<br>It may encourage others to seek help, and<br>It can be a healing and empowering experience for you, too. (12)
5 Selection of narratives		
5.1 Narrative selection	Reviewing submitted material	Every story submitted is reviewed by NAMI and all stories may not be posted on the blog. (8)
5.2 Narrative diversity	Seeking a diverse range of narratives	We intentionally included well-written memoirs that have inspired an entire field of writing and more common accounts that have not made the New York Times bestseller list. Sometimes, the well-written memoir is strikingly effective at describing the disorder and does so in an especially engaging manner. Other times, a simple account is basic but establishes a clear and meaningful everyday story. Our goal was to obtain a good mix of personal narratives for the reader. (3)
6 Editing of narratives		
6.1 Editing for clarity	Shortening, enhance flow, remove repetition	Technically, [working with narratives] involved editing them to a half or a third of their original length, cutting out the repetition within and between stories, and trimming details that were not strictly necessary to the flow and the sense. (18)
6.2 Editing for safety	Destroying identifying information	We have… changed names and other identifying information to make them anonymous, but have not changed the essence of them or their advice. (21)

7. Presentation of narratives		
7.1 Ordering	Order narrative by clinical diagnosis	The chapters in this book are organized by DSM-IV-TR categories so that students and other readers) can relate the first person accounts to what they are learning about diagnosis and mental disorders in the classroom and internship settings. (3)
	Order narratives to highlight mutual connections	I have ordered the chapters in a way that emphasises the similarities between some of the different stories on the basis of what caused each person's breakdown. (18)
7.2 Format	Allow a diversity of formats	Format: Story sharing may take any form of media, written (websites, booklets etc), live (presentations, live television broadcasts etc) or recorded (pre-recorded radio programming, DVD productions etc).<br>Each format has specific considerations which you need to be familiar with to ensure you can fully take part in any planning decisions with your supporters (explored in appendix 1: Specific format considerations). (3)
	Present narratives that conform to a specific format	Guest posts should be written for a US audience with American spellings. (11)<br><br>Guest posts should be between 700 and 1200 words (11)
8. Ethics and legality		
8.1 Consent	Establish clear consent for use (written or verbal)	The process of investing time to create a [consent] document, or spoken agreement, gives value to the experience for all involved. (3)
8.2 Ownership	Establish through formal written agreements	By submitting a post for publication on [charity]'s blog, you are confirming that the rights to any materials used are yours, and that any sources are credited as necessary. You are also granting [charity], its licensees, assignees, and other successors-in-interest, all rights to the copy. We may reproduce your blog, or quotes from your blog, in public relations (PR), electronic media and promotional materials. (7)
9 Societal positioning		
	Position relative to public policy	A close inspection of the NSF for Mental Health Department of Health (1999) reveals no mention of recovery. However, it does emerge briefly (albeit quite upfront in the title) in "The Journey to Recovery" (Department of Health, 2001a) - this policy document was subtitled the government's vision for mental health care. There are four short paragraphs on recovery, stating that a more optimistic approach is needed with the vast majority of those using mental health services having real prospects of recovery' (p. 22). (17)

	Position relative to clinical language	Putting together a book of first person accounts of "mental illness" immediately calls into question what constitutes a mental illness. Although our book educates about and conforms to the Diagnostic and Statistical Manual of Mental Disorders, 4th edition, Text Revision (DSMIV-TR) categories, it should not be perceived as an endorsement of the DSM. (3)
